# Downregulation of connective tissue growth factor inhibits the growth and invasion of gastric cancer cells and attenuates peritoneal dissemination

**DOI:** 10.1186/1476-4598-10-122

**Published:** 2011-09-28

**Authors:** Cheng-Gang Jiang, Ling Lv, Fu-Rong Liu, Zhen-Ning Wang, Fu-Nan Liu, Yan-Shu Li, Chun-Yu Wang, Hong-Yan Zhang, Zhe Sun, Hui-Mian Xu

**Affiliations:** 1 Department of Surgical Oncology, The First Affiliated Hospital of China Medical University, Shenyang, Liaoning Province, China; 2 Department of Thoracic Surgery, The First Affiliated Hospital of China Medical University, Shenyang, Liaoning Province, China; 3 Department of Cell Biology, China Medical University, Shenyang, Liaoning Province, China

**Keywords:** Connective tissue growth factor, Stomach neoplasms, Cell proliferation, Invasiveness, Peritoneal dissemination

## Abstract

**Background:**

Connective tissue growth factor (CTGF) has been shown to be implicated in tumor development and progression. However, the role of CTGF in gastric cancer remains largely unknown.

**Results:**

In this study, we showed that CTGF was highly expressed in gastric cancer tissues compared with matched normal gastric tissues. The CTGF expression in tumor tissue was associated with histologic grade, lymph node metastasis and peritoneal dissemination (P < 0.05). Patients with positive CTGF expression had significantly lower cumulative postoperative 5 year survival rate than those with negative CTGF expression (22.9% versus 48.1%, P < 0.001). We demonstrated that knockdown of CTGF expression significantly inhibited cell growth of gastric cancer cells and decreased cyclin D_1 _expression. Moreover, knockdown of CTGF expression also markedly reduced the migration and invasion of gastric cancer cells and decreased the expression of matrix metalloproteinase (MMP)-2 and MMP-9. Animal studies revealed that nude mice injected with the CTGF knockdown stable cell lines featured a smaller number of peritoneal seeding nodules than the control cell lines.

**Conclusions:**

These data suggest that CTGF plays an important role in cell growth and invasion in human gastric cancer and it appears to be a potential prognostic marker for patients with gastric cancer.

## Introduction

Despite significant advances in cancer research, cancer remains a worldwide health problem and mortality due to cancer remains high [1]. Gastric cancer is the second leading cause of cancer-related death in the world while there appears to be a decreasing trend in occurrence, notably in Western countries; it is still commonly reported in China and Japan [2,3]. Even though the prognosis of patients with advanced gastric cancer seems to have improved as a result of the standardization of surgical techniques and recent advances in chemotherapy, the 5-year postoperative survival rate remains low [4,5]. Peritoneal metastasis is the most common and significant cause of mortality after surgery for gastric cancer [6,7]. However, the mechanisms of peritoneal metastasis have not been clearly defined.

Connective tissue growth factor (CTGF), also known as CCN2, is a member of the CCN family, including cysteine-rich protein 61 (Cyr61), also known as CCN1, and nephroblastoma-over expressed gene (Nov), also known as CCN3, as well as Wisp-1/elm1 (CCN4), Wisp-2/rcop1 (CCN5) and Wisp-3 (CCN6) [8,9]. CTGF is believed to be a multifunctional signaling modulator involved in a wide variety of biologic or pathologic processes, such as angiogenesis, osteogenesis, fibrosis in kidneys and skin, and tumor development [10-12]. Although the role of CCN2 in normal tissue fibrosis has been well-studied [13], the function of CCN2 in cancer is not as well-understood. Interestingly, CCN2 has been identified as an oncogene in a variety of cancer types but is considered a tumor-suppressor gene in other forms of cancer [14]. Overexpression of CTGF is found in prostate cancer [15], gliomas [16], breast cancer [17], and adult acute lymphoblastic leukemia [18]. Increased CTGF expression has been associated with progression of cervical tumors [19], and esophageal squamous cell carcinoma [20]. Conversely, in lung adenocarcinomas [21] and colon cancers [22], overexpression of CTGF inhibits invasion and metastasis of the cancer cells both in vitro and in vivo.

Clinical studies have shown that overexpression of CTGF was significantly correlated with lymph node metastasis and poor prognosis in patients with gastric cancer [23,24]. However, the precise role of CTGF in gastric cancer is still unknown. In this study, we detected CTGF expression in gastric cancer tissues. We found that CTGF was overexpressed in gastric cancer, and its expression was associated with aggressive behavior of gastric cancer. We then used siRNA technology to knockdown endogenous CTGF expression in gastric cancer cells. We demonstrated that downregulation of CTGF inhibited the growth and invasion of gastric cancer in vitro and attenuated peritoneal dissemination in vivo. Our study strongly highlights the significance of CTGF in the growth and invasion of gastric cancer, and may provide a therapeutic target in gastric cancer.

## Materials and methods

### Reagents

Dimethyl sulfoxide (DMSO) and trypsin were purchased from Sigma (St. Louis, MO, USA). DMEM, streptomycin and other cell culture supplies were from GIBCOBRL (Grand Island, NY, USA). Fetal bovine serum was from Hyclone (Logan, UT, USA). MTT [3-(4, 5-dimethylthiazol-2-yl)-2, 5-diphenyltrazolium bromide] was obtained from Fluka (Ronkonkoma, NY, USA). CTGF, cyclin D1, matrix metalloproteinase (MMP)-2, MMP-3, MMP-9 and GAPDH primary antibody, as well as second antibody Rhodamine (TRITC)-conjugated affinipure goat anti-mouse IgG were obtained from Santa Cruz Biotechnology (Santa Cruz, CA, USA). CTGF ELISA kit was purchased from R&D (Minneapolis, MN, USA). Trizol and Lipofectamine 2000 were purchased from Invitrogen (Carlsbad, CA, USA). SYBR^@^Primescript™ RT-PCR kit was from Takara Biotechnology, Japan.

### Tissue specimens and immunohistochemical staining

Tumor specimens were obtained from 110 patients with gastric cancer who underwent surgery at the Department of Surgical Oncology, First Affiliated Hospital of China Medical University during the period 2003-2005. All patients underwent gastrctomy, and their clinical and pathological data were available. Normal stomach tissues were taken from the matched distal resected margin of gastric cancer samples. All surgical specimens were examined by experienced pathologists and the distal resected margin was tumor-free. The fresh tissues were cut into small pieces, snap-frozen in liquid nitrogen immediately, and stored at -80°C until protein extraction. The study protocol was reviewed and approved by the Ethics Committee of China Medical University.

For immunohistochemical staining, 4 μm histological sections were deparaffinized with xylene and rehydrated through a graded series of alcohol. The sections were then boiled for 10 min in 0.01 M citrate buffer and endogenous peroxidase was blocked by incubation in 0.3% H_2 _O_2 _in methanol for 30 min. Nonspecific binding was blocked by incubating slides with normal goat serum for 30 min at room temperature. The sections were incubated overnight at 4°C with 1:50 dilution of CTGF primary antibody. The sections were exposed to biotin-labeled secondary antibody for 1 h, to a streptavidin-peroxidase reaction system, and then developed with DAB- H_2 _O_2 _. Staining was scored on the following scale: 0, no staining; 1+, minimal staining; 2+, moderate to strong staining in at least 20% of cells; 3+, strong staining in at least 50% of cells. Cases with 0 or 1+ staining were classified as negative, and cases with 2+ or 3+ staining were classified as positive.

### Cell Culture

Human gastric cancer cell lines, MKN-45, MKN-1, AGS, SGC7901, BGC823 and MGC803 were obtained from the Department of Cell Biology, China Medical University, China. They were cultured in DMEM containing 10% fetal bovine serum, 100 U/ml of penicillin, 100 ug/ml of streptomycin at 37°C in a humidified atmosphere of 5% CO_2. _The cells were dislodged using 0.25% trypsin and 0.02 mol/L EDTA in PBS for subculture.

### Construction of CTGF knockdown stable cell lines

Two small interfering RNA (siRNA) oligonucleotides were synthesized to target two different regions in the CTGF cDNA: GTGCATCCGTACTCCCAAA (PSC1) and GCTAAATTCTGTGGAGTAT (PSC2). They were cloned into the siRNA expression vector pGCsilencer™U6.Neo.GFP. The nonspecific siRNA was used as a negative control (PSNC). The siRNA expression plasmids were transfected into SGC7901 cells using Lipofectamine 2000. The cells were screened with G418 (800 ug/ml), and the colonies were picked after 3 weeks, determined by RT-QPCR and Western blot. Cells transfected with PSC1, PSC2 or PSNC were designated PSC1 cells, PSC2 cells or PSNC cells, respectively.

### Real-Time Quantitative Polymerase Chain Reaction (RT-QPCR)

Total RNA was isolated from cell pellets using Trizol reagent. Total RNA (1 ug) was converted to cDNA using a RT (reverse transcriptase) reaction kit. Real-time PCR was performed using Mx3000P real-time PCR system according to the manufacturer's instruction and SYBR^® ^Premix ExTaq as a DNA specific fluorescent dye. PCR was carried out for 40 cycles of 95°C for 5 s and 60°C for 40 s. Primer sequences are shown in table 1. The threshold cycle (Ct) was obtained and relative quantities were determined for each sample normalized to GAPDH. Expressions of mRNA were calculated using the ΔΔCt method [25].

**Table 1:** PCR Primer Sequences

Gene	Primer Sequences (5'-3') Forward and Reverse	Product (bp)
CTGF	CTTGCGAAGCTGACCTGGAA AAAGCTCAAACTTGATAGGCTTGGA	90
MMP-2	ATGACATCAAGGGCATTCAGGAG TCTGAGCGATGCCATCAAATACA	135
MMP-3	GGGTGAGGACACCAGCATGA CAGAGTGTCGGAGTCCAGCTTC	178
MMP-9	TCCCAGACCTGGGCAGATTC GCAAAGGCGTCGTCAATCAC	124
Cyclin D1	GATGCCAACCTCCTCAACGAC CTCCTCGCACTTCTGTTCCTC	171
GAPDH	GCACCGTCAAGGCTGAGAAC TGGTGAAGACGCCAGTGGA	138

### Western blot analysis

Tissues or cells were lysed in RIPA buffer supplemented with protease inhibitor mixture for 30 min at 4°C. The cell lysates were then sonicated briefly and centrifuged (14,000 g at 4°C) for 15 min to remove insoluble materials. Equal amounts of protein were separated by SDS-PAGE and transferred to a PVDF membrane. Membranes were blocked with 5% nonfat dry milk and then incubated with first antibody, followed by horseradish peroxidase-conjugated secondary antibody. Protein bands were visualized by ECL chemiluminescence method.

### Immunofluorescence and Confocal imaging

The cells on Lab-Tek tissue culture chamber slides were fixed in cold 100% methanol for 10 min, and then blocked with normal goat serum for 30 min. The cells were incubated with the primary antibody overnight at 4°C, washed 3 times in PBT (PBS with 1‰ Triton X-100), and then incubated with second antibody conjugated with Rhodamine. The DNA dye DAPI was used to stain the DNA. Cells were imaged on a Leica SP2AOBS confocal microscope.

### Conditioned medium (CM) collection and Enzyme-linked immunoassay (ELISA)

3 × 10^5 ^cells were seeded in 100 mm tissue culture dish with DMEM containing 10% fetal bovine serum for 2 days. Then the cells were washed twice with PBS and incubated with 5 ml of serum free DMEM. 48 h later, the conditioned medium was collected and centrifuged at 2000 g for 5 min, passed through filters (pore size, 0.45 um) and stored at -80°C until use.

The levels of CTGF in the conditioned media from gastric cancer cell lines were measured using human Quantikine ELISA kit following the manufacturer's instructions.

### MTT proliferation assay

The capability of cellular proliferation was assessed using MTT [3-(4, 5-dimethylthiazol-2-yl)-2, 5-diphenyltrazolium bromide] assay. Approximately 5 × 10^3 ^cells were seeded into 96-well culture plates and cultured in serum free DMEM for 24, 48, 72, and 96 h, respectively. Then cells were incubated with 20 μl MTT (10 mg/ml) for 4 h at 37°Cand 200 μl DMSO was pipetted to solubilize the formazan product for 20 min at room temperature. The optical density (OD) was determined using a spectrophotometer (Bio-Rad) at a wavelength of 570 nm.

### Colony formation assay

5 × 10^2 ^cells suspended in 2 ml of 0.3% agarose DMEM medium containing 10% fetal bovine serum were plated in 6-well plates on the top of existing 0.6% bottom agarose with the same medium. The plates were incubated at 37°C in a 5% CO_2 _incubator. After three weeks, cell colonies > 0.1 mm in diameter were counted under a microscopic field.

### Cell invasion assay

The invasion was determined by an invasion chamber assay. Cells (2 × 10^4^) were seeded onto the top chamber of a 24-well matrigel-coated micropore membrane filter with 8 μm pores and the bottom chamber was filled with 0.5 ml of DMEM with 10% fetal bovine serum as a chemoattractant. After incubating for 24 h, non-invading cells (upper chamber) were gently removed by using a cotton-tipped swab and invading cells (lower chamber) were fixed using methanol and stained with trypan blue. The invasive ability was determined by the number of penetrating cells under a microscope at 200× magnification for 10 random fields in each well.

### Chamber migration assay

The method of in vitro migration assay was using a non-matrigel-coated 24-well Boyden chamber (8 μm, Millipore). Cells (2 × 10^4^) were seeded onto the inserts suspended in 0.2 ml of serum-free DMEM medium. Non-migrating cells were removed from the upper chamber of the filter after incubation for 24 h. Migrated cells were stained and quantified based on the procedure as described earlier. Triplicate assays were performed for each group of cells in invasion and migration assays, and the results are expressed as means ± SD.

### Gelatin zymography

MMP-2 and MMP-9 activity was determined by gelatin zymography as described previously [26]. In brief, after centrifugation the supernatant was separated and protein concentration determined; equal amounts of protein, added by sample buffer (Tris-HCl 1 M, pH 6.8, Sodium dodecyl sulphate (SDS) 2%, glycerol 10%) were applied to 7.5% SDS-polyacrylamide gel containing 1 mg/ml gelatine. After electrophoresis, SDS was removed from the gel by washing twice with 2.5% TritonX-100 for 1 h. After a brief rinse, the gel was incubated at 37°C for 18 h in buffer, pH 7.6, containing 100 mM Tris-HCl, 10 mM CaCl_2 _, 20 mM NaCl. The gel was stained with1% Coomassie Brilliant Blue R250 for 2 h and then treated with destaining solution (40% methanol, 10% acetic acid, 50% distilled water). Proteolytic activity was detected as clear bands against the background stain of undigested substrate in the gel.

### Animal study of peritoneal implantation

This experiment was conducted in accordance with the guideline issued by the State Food and Drug Administration (SFDA of China). The animals were housed and cared for in accordance with the guidelines established by the National Science Council of Republic China.

Female BALB/c nude mice, 35-40 days old and weighing 20-22 g, were supplied by Shanghai Slac Laboratory Animal Limited Company. The mice were kept under sterile conditions and fed a sterilized mouse diet and water. Animals were anaesthetized via inhalation of isoflurance. PSC1, PSC2, PSNC and SGC7901 cells (1 × 10^7 ^cells) were suspended in 0.5 ml DMEM and inoculated into the abdominal cavity of test mice. The mice were sacrificed six weeks later, and any disseminated nodules present on the mesentery and diaphragm were evaluated.

### Statistical analysis

All values in the text and figures are presented as mean ± SD. Overall survival rates were determined using Kaplan-Meier estimator, an event being defined as death for cancer correlated cause. The log-rank test was used to identify differences between the survival curves of different patients' groups. In univariate analysis, 2-tailed χ^2 ^tests for categorical variables and 2-tailed t test for continuous variables were used for statistical comparisons. Values of *p *< 0.05 were taken to show a significant difference between means.

## Results

### CTGF is overexpressed in gastric cancers and correlated with clinicopathological features of gastric cancer patients

**Figure 1:**
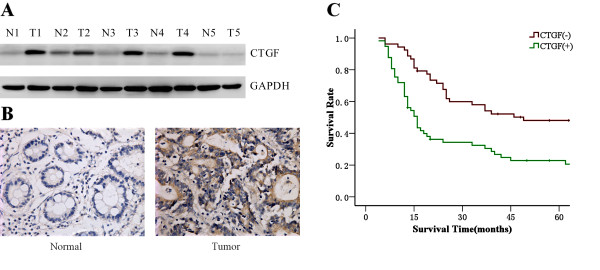
**Overexpression of CTGF in gastric cancer with worse prognosis**. **A**. Western blot analysis demonstrated the CTGF expression in gastric cancer tissues and matched distal normal tissues from five randomly selected gastric cancer patients. **B**. Immunohistochemistry results of CTGF expression in paired gastric cancer tissue samples. **C**. Kaplen-Meir survival curves for 110 patients with gastric cancer, grouped according to CTGF expression.

We determined the CTGF expression in gastric cancer tissues and matched distal normal tissues. Figure 1A illustrated CTGF expression in five randomly picked patients. Elevated levels of CTGF protein were found in human gastric cancer tissues compared with the paired normal tissues from the patients. This was also confirmed by immunohistochemical staining (Figure 1B). Moreover, in order to further investigate the correlation between expression of CTGF and clinicopathological features, 110 samples were used for examination with immunohistochemical staining. Statistical analysis revealed positive CTGF expression was significantly associated with histologic grade, lymph node metastasis and peritoneal dissemination compared with those patients with negative CTGF expression (Table 2). Positive CTGF expression was more frequently detected in cases of lymph nodes metastasis (P = 0.012). Levels of CTGF expression were increased significantly in undifferentiated gastric cancers compared with differentiated gastric cancers (P = 0.039). And the expression of CTGF protein was significantly correlated with the development of peritoneal dissemination of gastric cancer (P = 0.011). Calculation of the survival duration of the 110 involved patients by the Kaplan-Meier method revealed that the patients who featured CTGF-positive tumors demonstrated a shorter survival when compared with those patients who suffered from CTGF-negative tumors (Figure 1C, P < 0.001).

**Table 2:** Association between CTGF expression and clinicopathologic characteristics of patients with gastric cancer (n = 110)

	CTGF expression		
	Negative	Positive	P value
**Gender**			
Male	33	34	0.779
Female	20	23	
**Age (years)**			
≤ 65	35	40	0.642
> 65	18	17	
**Tumor size (cm)**			
< 5	26	25	0.585
≥ 5	27	32	
**Tumor location**			
Lower	42	37	0.413
Middle	5	8	
Upper	3	6	
Entire	3	6	
**Histologic grade**			
Differentiated	26	17	0.039*
Undifferentiated	27	40	
**Lauren grade**			
Intestinal	28	21	0.108
Diffuse	25	36	
**Lymph node metastasis**			
Negative	23	12	0.012*
Positive	30	45	
**TNM stage**			
I	11	8	0.080
II	15	9	
III	20	22	
IV	7	18	
**Hepatic metastasis**			
Negative	50	55	0.588
Positive	3	2	
**Peritoneal dissemination**			
Negative	50	44	0.011*
Positive	3	13	

*P < 0.05; CTGF, connective tissue growth factor.

### Expression of CTGF in human gastric cancer cell lines and siRNA-mediated silence

**Figure 2:**
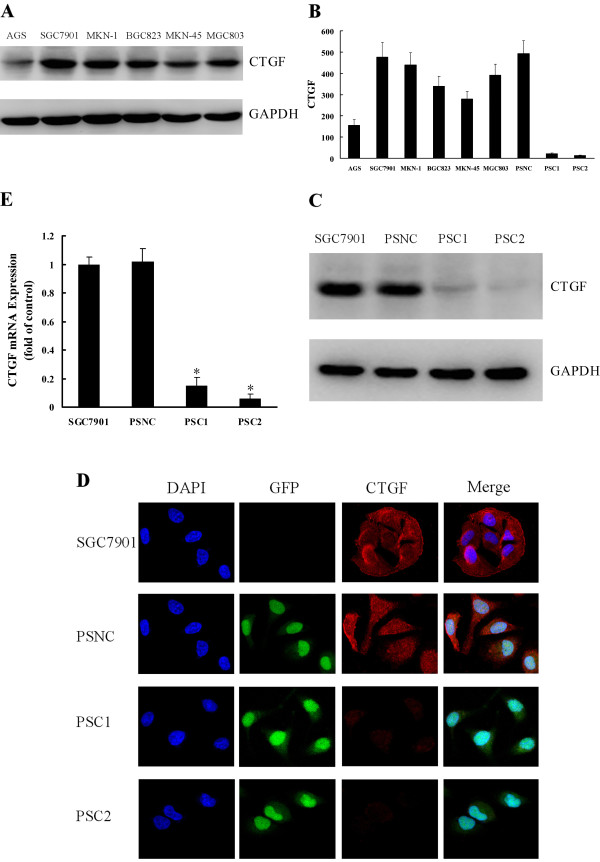
**Expression of CTGF in gastric cancer cell lines and knockdown of CTGF by siRNA**. **A**. Western blot showing the expression of CTGF in 6 gastric cancer cell lines. GAPDH served as protein loading control. **B**. CTGF in conditioned media of 6 gastric cancer cell lines and stable transfected cells was analyzed by ELISA. Results are expressed as pg/ml/1 × 10^6^cells (mean ± SD, n = 4). **C**. Western blot analysis of CTGF protein expression in SGC7901, PSNC and CTGF knockdown stable cell lines (PSC1 and PSC2). **D**. Immunofluorescence analysis of CTGF expression. **E**. RT-QPCR showing CTGF mRNA levels in SGC7901, PSNC and CTGF knockdown stable cell lines (PSC1 and PSC2). Data are expressed as a fold change relative to control (control is SGC7901). Values are given as mean ± SD of three experiments. *P < 0.05 as compared to control.

First, we examined CTGF expression in six gastric cancer cell lines (MKN-45, MKN-1, AGS, SGC7901, BGC823 and MGC803) by Western blot. CTGF was detected in all cell lines evaluated, and with SGC7901 cells expressing the highest level (Figure 2A). Therefore, SGC7901 cells were selected as the model for the subsequent function studies. Because biologically active CTGF is both secreted and expressed in the cytoplasm [8,27], we also measured the level of secreted CTGF in the conditioned media of these gastric cancer cell lines by ELISA, which was coincided with the level of CTGF in the cytoplasm of each cell line (Figure 2B).

To study the function of CTGF in SGC7901 cells, the CTGF knockdown stable cell lines were used to analyze the silencing effect. As shown in Figure 2B, the level of secreted CTGF in the conditioned medium was significantly decreased in the siRNA-stable transfected cells compared to control. Western blot and immunofluorescence staining showed that expression of CTGF protein in the cytoplasm decreased markedly in the CTGF knockdown stable cell lines (Figure 2C, D). Furthermore, the expression of CTGF mRNA was also significantly decreased in the CTGF knockdown stable cell lines (Figure 2E).

### Knockdown of CTGF expression inhibits the growth of gastric cancer cells

**Figure 3:**
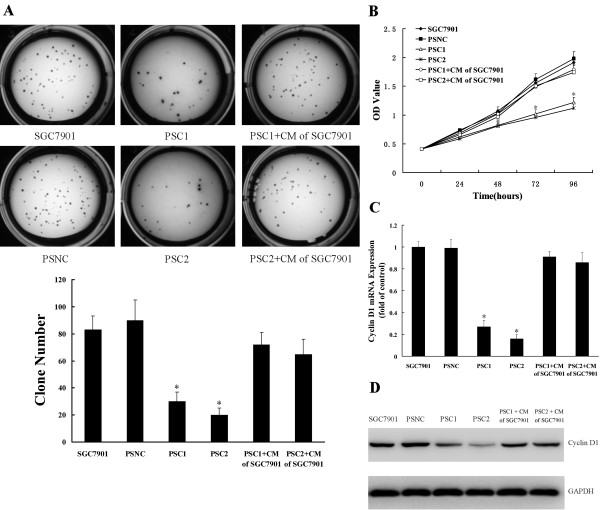
**Knockdown of CTGF expression inhibits the growth of gastric cancer cells**. **A**. Colony formation assay. **B**. MTT proliferation assay. **C**. Knockdown of CTGF down regulated the mRNA level of cyclin D1. **D**. Knockdown of CTGF down regulated the protein level of cyclin D1. PSC1/PSC2+CM of SGC7901: PSC1 or PSC2 cells were incubated with conditioned medium (CM) of SGC7901. All results were reproducible in three independent experiments. *P < 0.05 as compared to control (control is SGC7901).

The colony formation assay was used to evaluate the growth of the cells in which CTGF was silenced. As shown in Figure 3A, CTGF knockdown stable cells (PSC1 and PSC2) formed significantly fewer colonies on soft agar compared to SGC7901 and PSNC cells (83 ± 10, 90 ± 15 versus 30 ± 7 and 20 ± 5, respectively). To further test the negative effect of CTGF knockdown on gastric cancer cell growth, MTT assay was performed and growth curves were generated (Figure 3B). As shown by the curves, both PSC1 and PSC2 cells proliferated slower than PSNC cells and SGC7901 cells during the first 96 h after the cells were plated. The dramatic reduction of colony formation and growth of CTGF silenced cells suggested CTGF suppression might negatively regulate gastric cancer cell growth. RT-QPCR showed that mRNA levels of cell cycle related protein cyclin D1 were down regulated in the two CTGF knockdown stable cell lines compared with PSNC and SGC7901 (Figure 3C). Consistent with this result, we observed a marked reduction of cyclin D1 protein expression in CTGF knockdown cells PSC1 and PSC2 (Figure 3D). Interestingly, treatment with conditioned medium of SGC7901 which secreted a large amount of CTGF was able to rescue the cyclin D1 downregulation and restore the cell proliferation in CTGF knockdown stable cells. These data indicated that CTGF suppression might negatively regulate gastric cancer cell growth and decrease cyclin D1 expression.

### Knockdown of CTGF expression inhibits the migration and invasion of gastric cancer cells

**Figure 4:**
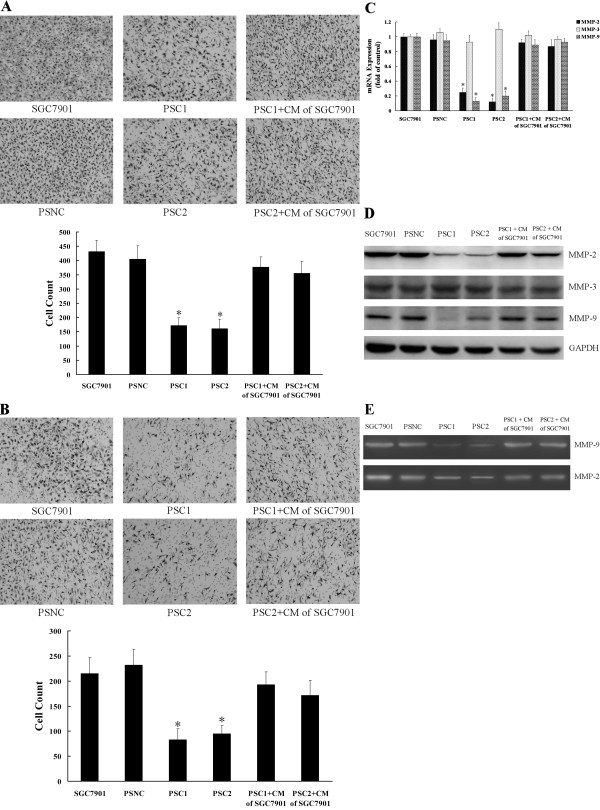
**Knockdown of CTGF expression inhibits the migration and invasion of gastric cancer cells**. **A**. Cell migration assay. **B**. Cell invasion assay. Migration and invasion cells were fixed and stained, and representative fields were photographed. For quantification, the cells were counted in 10 random fields under a light microscope (×200). Triplicate assays were performed for each group of cells in invasion and migration assays, and the results are expressed as means ± SD. **C**. RT-QPCR was done for analysis the mRNA expression of MMP-2, MMP-3, and MMP-9 in the CTGF knockdown stable cell lines and control cells. Bars represent the mean ± SD of three experiments. **D**. The protein levels of MMP-2, MMP-3, and MMP-9 were detected by Western blot. GAPDH served as protein loading control. **E**. Gelatin zymography analysis for the activities of MMP-2 and MMP-9. PSC1/PSC2+CM of SGC7901: PSC1 or PSC2 cells were incubated with conditioned medium (CM) of SGC7901.*P < 0.05 as compared to control (control is SGC7901).

Cell migration and invasion are critical processes in tumor metastasis. We investigated cell migration by non-matrigel-coated Boyden chamber and cell invasion by matrigel-coated invasion chamber assays, respectively. In the migration assays (Figure 4A), migration rates of the PSC1 and PSC2 cells were significantly decreased when compared to control (P < 0.05). As shown in Figure 4B, CTGF knockdown also markedly reduced cell invasion properties when compared to control. Cell invasion rates of PSC1 and PSC2 cells were decreased by 61.4% and 55.8%, respectively. RT-QPCR and Western blot showed that MMP-2 and MMP-9 were down regulated in the two stable clones compared with control cells (Figure 4C; D). In contrast, MMP-3 didn't significantly change both in mRNA and protein levels. Furthermore, zymography analysis showed the activities of both MMP-2 and MMP-9 in the siRNA-stable transfected cells were significantly lower than those in the control cells (Figure 4E). Interestingly, treatment with conditioned medium of SGC7901 which secreted a large amount of CTGF induced the re-expression of MMP-2 and MMP-9 and restored the migration and invasion of the CTGF knockdown stable cells. These results suggested that knockdown of CTGF expression reduced the migration and invasion of gastric cancer cells and decreased the expression of MMP-2 and MMP-9.

### Downregulation of CTGF inhibits peritoneal dissemination of gastric cancer in vivo

**Figure 5:**
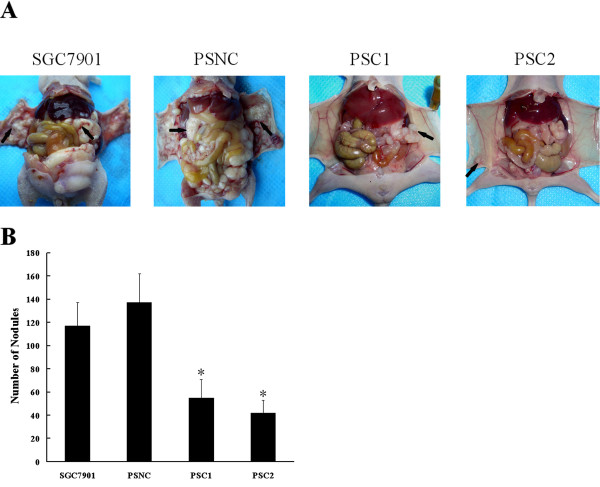
**Knockdown of CTGF inhibits gastric cancer SGC7901 xenograft peritoneal dissemination**. SGC7901, PSNC and CTGF knockdown stable cell lines (PSC1 and PSC2) were injected intraperitoneally as described under "Material and Methods". 6 weeks later, the mice were sacrificed, photographed, dissected and any disseminated nodules present on the mesentery and diaphragm were counted. **A**. The photograph of nude mice with peritoneal dissemination from each group. **B**. The disseminated nodules were evaluated. Each bar represents the mean ± SD (n = 5 for each group). *P < 0.05 as compared to control (control is SGC7901).

To explore the effects of CTGF on the peritoneal dissemination of gastric cancer cells in vivo, we inoculated different cells into nude mice. SGC7901 cells, control stable cell line (PSNC), and CTGF knockdown stable cells (PSC1 and PSC2) were injected into four separate groups of nude mice. As consequence of such treatment, the measurable suppression of peritoneal dissemination in mice injected with CTGF knockdown stable cells as compared with those injected with SGC7901 cells or PSNC cells was noted (Figure 5A). Quantitatively, 117 ± 20 disseminated nodules were noted for test mice inoculated with SGC7901 cells and 137 ± 26 disseminated nodules were noted for mice inoculated with PSNC cells. In contrast, significant fewer disseminated nodules were able to be observed for mice injected with CTGF knockdown stable cells (Figure 5B).

## Discussion

The most extensive literature to date regarding CTGF defines its role in wound-healing and fibrotic disease. Recently, several studies implicate CTGF in tumor development and tumor cell survival [28-30]. Nonetheless, the exact role of CTGF in tumor progression is not definite, and the function of CTGF in tumor cell biology of gastric cancer has not been thoroughly investigated. To address these issues, we evaluated CTGF expression with regard to possible direct correlations with cell growth and invasion of gastric cancer cells. Moreover, we further investigated the effects of CTGF on peritoneal dissemination of gastric cancer cells in vivo.

In this study, our results showed that CTGF was highly expressed in gastric cancer tissues compared with matched normal gastric tissues. The expression of CTGF in undifferentiated gastric cancer was significantly higher than those in differentiated gastric cancer. The CTGF expression in tumor tissue was associated with lymph node metastasis and peritoneal dissemination. Furthermore, patients with positive CTGF expression had significantly lower cumulative postoperative 5 year survival rate (22.9%) than those with negative CTGF expression (48.1%, Figure 1C). These results suggested that CTGF might be involved in progression and metastasis of gastric cancer. Moreover, CTGF might be a useful prognostic marker.

Several recent studies have revealed that CTGF regulate cell growth in esophageal cancer cells and pancreatic cancer cells [20,30]. However, little is known about the effect of CTGF expression on cell growth of gastric cancer cells. Our results showed that CTGF suppression resulted in inhibition of cell proliferation and clonogenic growth. Changes in cell growth might be key factors in regulating cancer progression [31]. Therefore, our results indicate a possible role for CTGF in gastric cancer development. We also found that expression of cyclin D1 decreased with suppression of CTGF. It is well known that cyclin D1 is important in the development and progression of numerous cancers [32,33]. Cyclin D1 plays a crucial role in the progression of cell cycle and determines mitochondrial function and size [34-36]. Cyclin D1 regulates cell cycle progression, explaining in part how CTGF influences the growth of gastric cancer cells.

Cancer metastasis is a major cause of morbidity in cancer patients. Cancer metastasis consists of multiple sequential steps; invasion is one of the most characteristic steps during the cascade of metastasis. Many studies have demonstrated the importance of invasion in the early stages of metastasis [37,38]. In this study, stable transfection with CTGF siRNA into human gastric cancer cells could obviously inhibit cell invasion and migration ability of SGC7901 (Figure 4A; B), which suggested that CTGF might be involved in metastasis of gastric cancer. The initial step of tumor cell invasion is characterized by the breakdown of the base membrane, a process known to be dependent on type IV collagen-degrading enzymes, mainly MMP-2 and MMP-9 [39]. The activation of MMP-2 and MMP-9 is in a tumor-specific manner and correlates with metastatic abilities and poor prognosis [40,41]. The data showed that the expression levels of MMP-2 and MMP-9 significantly decreased in the CTGF knockdown stable cell lines (PSC1 and PSC2), suggesting that this downregulation of MMP-2 and MMP-9 contributed to the reduced invasion of the CTGF knockdown stable gastric cancer cells.

Peritoneal dissemination is not only the most frequent pattern of gastric cancer recurrence, but it is also a major cause of death among advanced gastric cancer patients [42]. Although the presence of peritoneal metastasis reveals a strong impact for patient prognosis, the molecular mechanisms by which gastric cancer cells actually acquire the ability to undergo peritoneal dissemination remains to be clarified. In the present study, the CTGF expression in tumor tissue was associated with peritoneal dissemination. And the downregulation of CTGF was resulted in the inhibition of cell growth, migration and invasion of gastric cancer cells. Since cell growth and invasion are the critical steps of peritoneal dissemination, we investigated the effects of CTGF on the peritoneal dissemination of gastric cancer cells in vivo. We found CTGF knockdown stable cell lines (PSC1 and PSC2) had significantly diminished peritoneal metastatic ability compared with control cells. These results indicated CTGF might play an important role in the peritoneal dissemination of gastric cancer.

## Conclusions

In summary, overexpression of CTGF was associated with progression, metastasis and prognosis of gastric cancer. With siRNA technology, we showed that downregulation of CTGF expression could inhibit the cell growth and invasion of gastric cancer cells in vitro. Furthermore, downregulation of CTGF expression could attenuate peritoneal dissemination of gastric cancer cells in vivo. These data provide a sound scientific rationale for further investigation into targeting CTGF in gastric cancer.

## Competing interests

The authors declare that they have no competing interests.

## Authors' contributions

CGJ, LL, FRL, YSL and CYW carried out the experimental work. ZNW and FNL provided data analysis. HMX and ZS provided tumor samples, clinical information, and histopathological analysis. CGJ, HMX, ZNW, LL and HYZ designed the study and participated in writing the paper. All authors read and approved the manuscript.
